# Biotransformation of Benzoate to 2,4,6-Trihydroxybenzophenone by Engineered *Escherichia coli*

**DOI:** 10.3390/molecules26092779

**Published:** 2021-05-08

**Authors:** Anuwatchakij Klamrak, Jaran Nabnueangsap, Natsajee Nualkaew

**Affiliations:** 1 Faculty of Pharmaceutical Sciences, Khon Kaen University, Khon Kaen 40002, Thailand; anuwat_kla@yahoo.com; 2 Salaya Central Instrument Facility RSPG, Mahidol University, Nakhon Pathom 73170, Thailand; jaran.nab@mahidol.ac.th

**Keywords:** *Escherichia coli*, biotransformation, sodium benzoate, benzoate-CoA ligase, benzophenone synthase, 2,4,6-trihydroxybenzophenone, structural annotation

## Abstract

The synthesis of natural products by *E. coli* is a challenging alternative method of environmentally friendly minimization of hazardous waste. Here, we establish a recombinant *E. coli* capable of transforming sodium benzoate into 2,4,6-trihydroxybenzophenone (2,4,6-TriHB), the intermediate of benzophenones and xanthones derivatives, based on the coexpression of benzoate-CoA ligase from *Rhodopseudomonas palustris* (BadA) and benzophenone synthase from *Garcinia mangostana* (GmBPS). It was found that the engineered *E. coli* accepted benzoate as the leading substrate for the formation of benzoyl CoA by the function of BadA and subsequently condensed, with the endogenous malonyl CoA by the catalytic function of BPS, into 2,4,6-TriHB. This metabolite was excreted into the culture medium and was detected by the high-resolution LC-ESI-QTOF-MS/MS. The structure was elucidated by in silico tools: Sirius 4.5 combined with CSI FingerID web service. The results suggested the potential of the new artificial pathway in *E. coli* to successfully catalyze the transformation of sodium benzoate into 2,4,6-TriHB. This system will lead to further syntheses of other benzophenone derivatives via the addition of various genes to catalyze for functional groups.

## 1. Introduction

Benzophenone comes with a variety of structures and pharmacological activities [1]. The most important sources of biologically active benzophenones are plants in the Clusiaceae and Hypericaceae, which have been a huge benefit on human health [1,2]. Currently, more than 300 benzophenones have been isolated and structurally characterized [1,2,3]. 2,4,6-trihydroxybenzophenone (2,4,6-TriHB) (**5**) serves as a basic benzophenone skeleton (BBS) that undergoes poststructural modifications, including hydroxylation, cyclization, and prenylation, yielding a myriad range of prenylated benzophenone derivatives [2,4,5,6]. From the literature, polyprenylated benzophenones possess various potent biological activities, including cytotoxicity [7,8], antibacterial [9,10], antifungal [11,12], antioxidant [13,14], antiviral [15,16,17], antiparasitic [18,19], anti-inflammatory [20,21], and transcription regulation of lipid and glucose metabolism [22,23]. Chamuangone (**6**) is an example of a benzophenone derivative obtained from the leaves of *Garcinia cowa* (Clusiaceae) that exhibits antibacterial, antileishmania [24,25], and anticancer effects against lung adenocarcinoma (SBC3 and A549) and leukemia (K562 and K562/ADM) [26]. α-mangostin (**6**), from the fruit pericarps of *G. mangostana*, is also benzophenone’s derivative, and it exhibits potent antiproliferation effects on tumor cells and presents with potential application as anticancer and chemopreventive agents [27,28,29]. Generally, the bioactive benzophenones and xanthones could be prepared by purification from plant raw materials and chemical synthesis [2,30,31]. Both methods are not friendly to the natural environment, as they generate plenty of waste from organic solvents. Besides, reliance on natural resources is often limited by their growing time, rare distribution in some regions, and seasonal variation in their active contents, as well as the processes of purification, such as time, labor, and the organic solvents used [32]. Moreover, the chemical synthesis of those compounds is challenged by their structural complexity, in which multiple steps and a large number of starting precursors and solvents are required [33].

The engineering of microbes using whole-cell biotransformation to produce bioactive metabolites from low-priced substrates is more desirable than chemical synthesis and isolation from plant materials [34]. Microbial systems contain many benefits, i.e., a fast-growing rate, less chemical consumption, and ability to upscale production using bioreactors [35,36]. Notably, microbes also contain their native metabolisms (e.g., glycolytic and fatty acid syntheses) that could supply the precursors involved in polyketide synthesis, i.e., phenylalanine, tyrosine, ATP, CoASH, and malonyl-CoA (**4**), thereby reducing chemical consumption [37]. For these reasons, in the last few decades, *Escherichia coli* has been engineered as a biotransformation system to produce bioactive polyketides, such as stilbenoids, flavonoids, and curcuminoids, using phenylpropanoid acids (e.g., p-coumaric acid and cinnamic acid) as the fed precursors [38,39,40].

Biosynthetically, 2,4,6-TriHB (**5**) originated from a reaction between one starter unit of benzoyl CoA (**3**) and 3 extender units of malonyl-CoA (**4**) through the catalytic function of benzophenone synthase (BPS) [4,41]. The genes encoding for BPS have been isolated from plants in Hypericaceae, including *Hypericum sampsonii* (HsBPS), *H. androsaemum* (HaBPS), and *H. perforatum* (HpBPS) [42,43,44], and plants from Clusiaceae, such as *Garcinia mangostana* (GmBPS) [41]. Up to now, there have been two established strategies for synthesizing this compound, including total chemical synthesis and enzymatic reaction [4,41,42,43,44,45]. The first method utilizes phloroglucinol and benzonitrile as reactants under an acid catalysis condition that consists of multiple processes [45]. The latter one proceeds via the enzymatic reaction of benzophenone synthase with benzoyl-CoA (**3**) and malonyl-CoA (**4**) [4,42], which requires the expensive precursors benzoyl CoA (**3**) and malonyl-CoA (**4**). In a preliminary experiment, we attempted to produce 2,4,6-TriHB (**5**) by supplementing benzoyl-CoA (**3**) (lithium salts) into cultures of *E. coli* BL21(DE3)pLysS harboring plasmid pET22b-GmBPS; however, the experiment was unsuccessful because there were no putative mass peaks corresponding to 2,4,6-TriHB (**5**) (calc. *m*/*z*: 231.065185 [M + H]^+^) detected. A similar trend was found in an attempt to produce 2,4,6-TriHB (**5**) by feeding benzoyl-CoA (**3**) into *Saccharomyces cerevisiae* engineered to harbor HaBPS and acetyl-CoA carboxylase (ACC), which might be because the fed precursor was not able to penetrate across the yeast cell wall or because it was rapidly degraded by endogenous native acyl-CoA thioesterases before interacting with the HaBPS [46]. However, the production of 2,4,6-TriHB (**5**) through feeding benzoyl-CoA (**3**) to microbial cells is unsuitable from an economic point-of-view since a large amount of a high-priced substrate would need to be added into the medium culture. Therefore, this strategy is not practical to use in scaled-up production. To solve all of those limitations, engineered *E. coli* strains capable of synthesizing 2,4,6-TriHB (**5**) from low-priced substrates, e.g., sodium benzoate (**1**), needed to be established.

**Figure 1:**
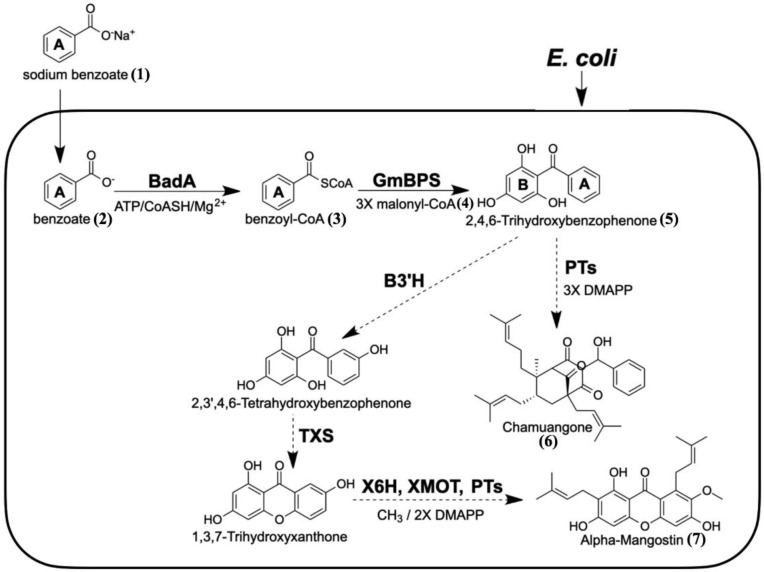
Proposed biosynthetic pathway of 2,4,6-TriHB (**5**) in *E. coli* via the coexpression of BadA and GmBPS (heavy lines). Sodium benzoate (**1**) was used as the fed precursor. The synthetic routes that might be extended from 2,4,6-TriHB (**5**) towards alpha-mangostin (**7**) and chamuangone (**6**) are shown (dotted lines). (BadA: benzoate-CoA ligase; GmBPS: G. mangostana benzophenone synthase; PTs: prenyltransferase; B3H: benzophenone 3-hydroxylase; TXS: trihydroxyxanthone synthase; X6H: xanthone 6-hydroxylase; XOMT: xanthone-O-methyltransferase; DMAPP: dimethylallyl diphosphate).

The benzoate-CoA ligase from *R. palustris* (BadA, GenBank accession no. L42322.1) is an enzyme that catalyzes the formation of benzoyl-CoA (**3**) from the benzoate (**2**), and it has been used in the metabolic engineering of soraphen A, an antifungal polyketide, in Streptomyces lividans [47,48,49]. This illustrates the potential application of BadA in coexpression with GmBPS (Genbank accession no. JF907623.1) to produce 2,4,6-TriHB (**5**) after supplementing sodium benzoate (**1**) in an *E. coli* culture (Figure 1). Hence, this study was conducted as “a proof-of-concept experiment” to establish the *E. coli* BL21(DE3)pLysS strain as a biotransformation system capable of producing 2,4,6-TriHB (**5**) from fed sodium benzoate (**1**), based on the strategies described by Tolia and Joshual-Tor, 2006 [50]. We found that *E. coli* BL21(DE3)pLysS engineered to carry plasmid pETDuet-BadA-GmBPS was able to produce 2,4,6-TriHB (**5**) from sodium benzoate (**1**) supplied into the culture. To ensure product formation, the experimental mass spectra for the putative 2,4,6-TriHB (**5**) (positive and negative ion modes) were further elucidated by using SIRIUS (version 4.5), the in silico tool designed for turning tandem mass spectra into the correct molecular formula [51,52]. Sirius is also integrated with the CSI:FingerID web service and the CANOPUS tool to provide more details about the chemical structure and compound classifications of the query subjects, thereby helping elucidate the chemical structure of the query metabolites [53,54,55]. Several factors that might have affected the yield of 2,4,6-TriHB (**5**) in the case of *E. coli* used as a biotransformation system are also discussed.

## 2. Results

### 2.1. Identification of 2,4,6-TriHB *(**5**)* by Using the High-Resolution LC-ESI-QTOF-MS/MS

**Figure 2:**
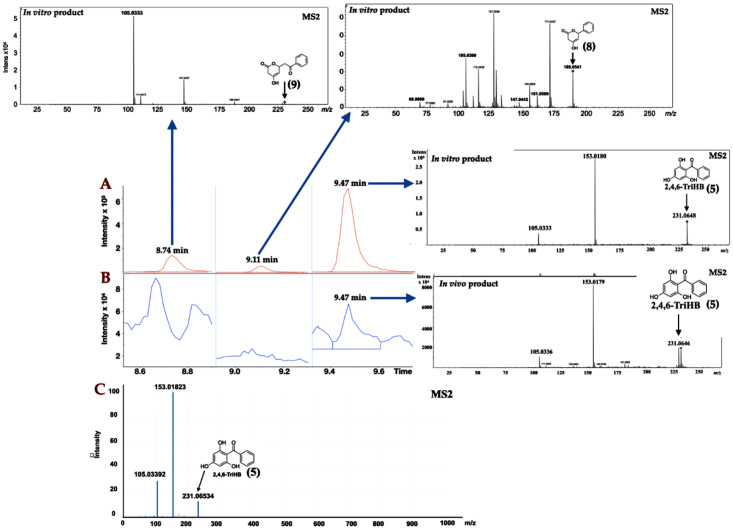
Identification of 2,4,6-TriHB (**5**) using the positive ion mode in LC-ESI-QTOF-MS/MS (CID of 20 eV) with multiple reaction monitoring (MRM) mode. (**A**) Selected regions of the total ion chromatogram (TIC) from the enzymatic products of GmBPS (red), which consisted of tetraketide lactone (**9**), triketide lactone (**8**), and 2,4,6-TriHB (**5**), with their unique MS/MS spectra that was obtained from the EICs (inserted pictures); (**B**) The regions of TIC of the secreted products from clones harboring pETDuet-BadA-GmBPS (blue) consisting only of the putative 2,4,6-TriHB peak (**5**) where the two ketide lactones (**8**, **9**) were absent; (**C**) The MS/MS spectra of the standard 2,4,6-TriHB (**5**) analyzed by the LC-ESI(+)-QFT technique, retrieved from the PubChem database (submitted by Megan Showalter, University of California, Davis).

The high-resolution LC-ESI-QTOF-MS/MS (both positive and negative ionization modes) with multiple reaction monitoring (MRM) was used for the identification of 2,4,6-TriHB (**5**) from the bioconversion of benzoate (**1**) by the clones carrying pETDuet-BadA-GmBPS. In this study, 2,4,6-TriHB (**5**) prepared from the in vitro reaction between benzoyl CoA (**3**) and malonyl-CoA (**4**), which was catalyzed by GmBPS, was used as the reference standard. There were typically 3 peaks: tetraketide lactone (**9**), triketide lactone (**8**), and the major peak, 2,4,6-TriHB (**5**), detected from the in vitro reaction as shown in Figure 2A. The culture extract showed the TIC region at the retention time of 9.47 min (Figure 2B), the molecular ion of *m*/*z* 231.0646 [M + H]^+^ (calc. *m*/*z*: 231.065185), and daughter ions with *m*/*z* 153.0.179 and 105.0336, which were identical to the enzymatically-derived 2,4,6-TriHB (**5**). The secreted product (9.47 min) also displayed an identical fragmentation profile to that of 2,4,6-TriHB (**5**), as reported in the PubChem database (https://mona.fiehnlab.ucdavis.edu/spectra/display/FiehnHILIC000683 (accessed on 25 April 2020) (Figure 2C). On the other hand, based on the elected ion chromatograms (EICs), there were no putative peaks corresponding to triketide lactone (**8**) or tetraketide lactone (**9**) detected from the culture medium of clones carrying pETDuet-BadA-GmBPS. Therefore, the secreted product eluted from the LC column at 9.47 min is believed to be 2,4,6-TriHB (**5**).

**Figure 3:**
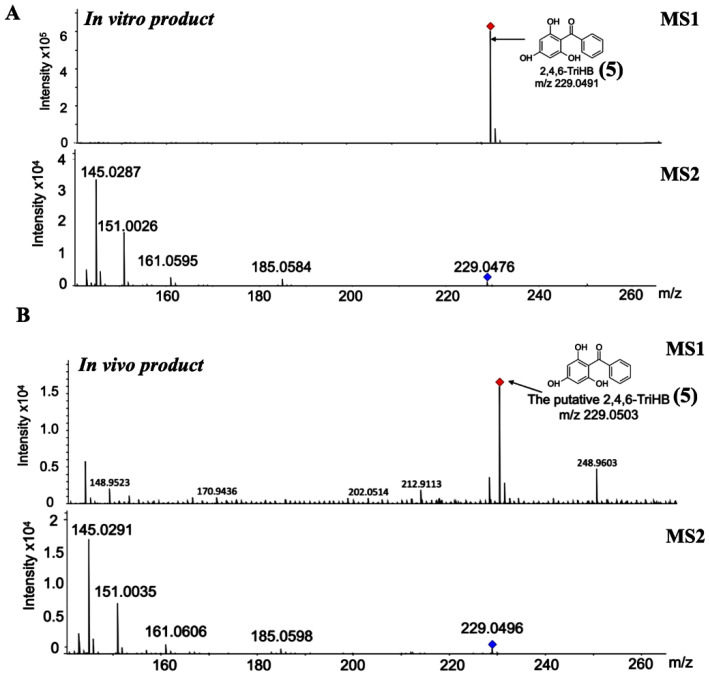
Identification of 2,4,6-TriHB (**5**) by using the high-resolution LC-ESI(−)-QTOF-MS/MS (CID of 35 eV). (**A**) The mass spectrum of 2,4,6-TriHB (**5**) (*m/z* 229.0491) with the MS/MS fragmentation pattern from the enzymatic reaction of GmBPS, where benzoyl-CoA (**3**) and malonyl-CoA (**4**) were the substrates. (**B**) The putative mass spectrum of 2,4,6-TriHB (**5**) (*m/z* 229.0503) and its fragmentation profile obtained from the bioconversion of benzoate (**2**) by *E. coli* BL21(DE3)pLysS harboring pETDuet-BadA-GmBPS.

The high-resolution LC-ESI-QTOF-MS/MS in negative ionization mode was used to confirm the existence of 2,4,6-TriHB (**5**) in the culture medium of clones carrying pETDuet-BadA-GmBPS by monitoring the molecular ion *m*/*z* 229.050632 [M − H]^−^ (computed by using the mass calculations tool). In the culture medium extract, the molecular ion with an *m*/*z* of 229.0503 was detected as expected (Figure 3B). This compound also exhibited an identical fragmentation pattern to that of the standard 2,4,6-TriHB (**5**) prepared from the enzymatic reaction of GmBPS, characterized by the product ions with *m*/*z* 145, 151, 161, and 185 (Figure 3A). The mass fragmentation profile was also in line with that of 2,4,6-TriHB (**5**) (*m*/*z* 229.0503) measured by LC-ESI-QFT in negative ion mode, which was deposited in the PubChem database (Figure S1). Therefore, these results confirmed that *E. coli* BL21(DE3)pLysS was engineered to carry pETDuet-BadA-GmBPS capable of synthesizing 2,4,6-TriHB (**5**) from the fed benzoate (**1**).

### 2.2. Localisation of 2,4,6-TriHB *(**5**)*

**Figure 4:**
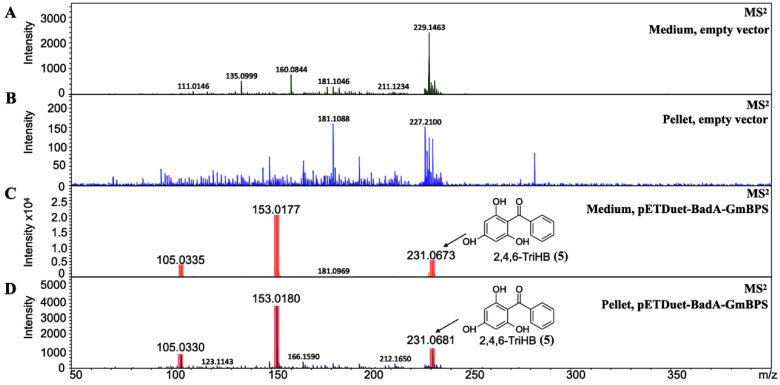
The identification of 2,4,6-TriHB (**5**) from the culture medium and pellets by using high-resolution LC-ESI (+)-QTOF-MS/MS (CID of 20 eV). (**A**) No MS/MS spectra of 2,4,6-TriHB (**5**) were detected from the medium extract of clones harboring pETDuet-1 empty vector (control). (**B**) No MS/MS spectra of 2,4,6-TriHB (**5**) was found in the pellet extract of clones harboring pETDuet-1 empty vector. (**C**) The MS/MS spectra of 2,4,6-TriHB (**5**) (red highlights) from the medium of clones harboring pETDuet-BadA-GmBPS. (**D**) The MS/MS spectra of 2,4,6-TriHB (**5**) (red highlights) from the pellet of clones carrying pETDuet-BadA-GmBPS.

Intracellular accumulation and extracellular secretion were investigated to gain more knowledge on 2,4,6-TriHB biosynthesis in *E. coli*. Based on the data measured by LC-ESI(+)-QTOF-MS/MS, 2,4,6-TriHB (**5**) was detected both in the pellets and medium (Figure 4C,D), but this compound was absent in *E. coli* BL21(DE3)pLysS-carried pETDuet-1 vector (Figure 4A,B). Based on the mass data retrieved from the TICs, the peak areas of the putative 2,4,6-TriHB (**5**) measured from the pellets and the culture medium were 3576.1 and 36,747.0, respectively. The results indicated that this compound was efficiently secreted into the culture medium, which had levels of the compound approximately 10.28-fold higher than that of the cell pellets.

### 2.3. Structural Confirmation of 2,4,6-TriHB *(**5**)* by Using SIRIUS

#### Molecular Formula Annotation from the Positive Mode Mass Data 

Based on the Sirius user manual, high-accuracy mass data with a mass deviation inside the range of 20 ppm is required before the annotation process to obtain reliable results. Therefore, the quality of the putative mass spectrum of 2,4,6-TriHB (**5**) (*m*/*z* 231.0646 [M + H]^+^) was evaluated by using the mass error calculation tool provided on a web-based service (https://warwick.ac.uk/fac/sci/chemistry/research/barrow/barrowgroup/calculators/mass_errors/ (accessed on 20 December 2020), by comparing it against the theoretical *m*/*z* of 2,4,6-TriHB (**5**) (*m*/*z* 231.065185 [M + H]^+^). The analysis confirmed that the putative mass spectrum exhibited an insignificant mass deviation (with only −1.666196 ppm error), allowing for further elucidation by the Sirius tool.

**Figure 5:**
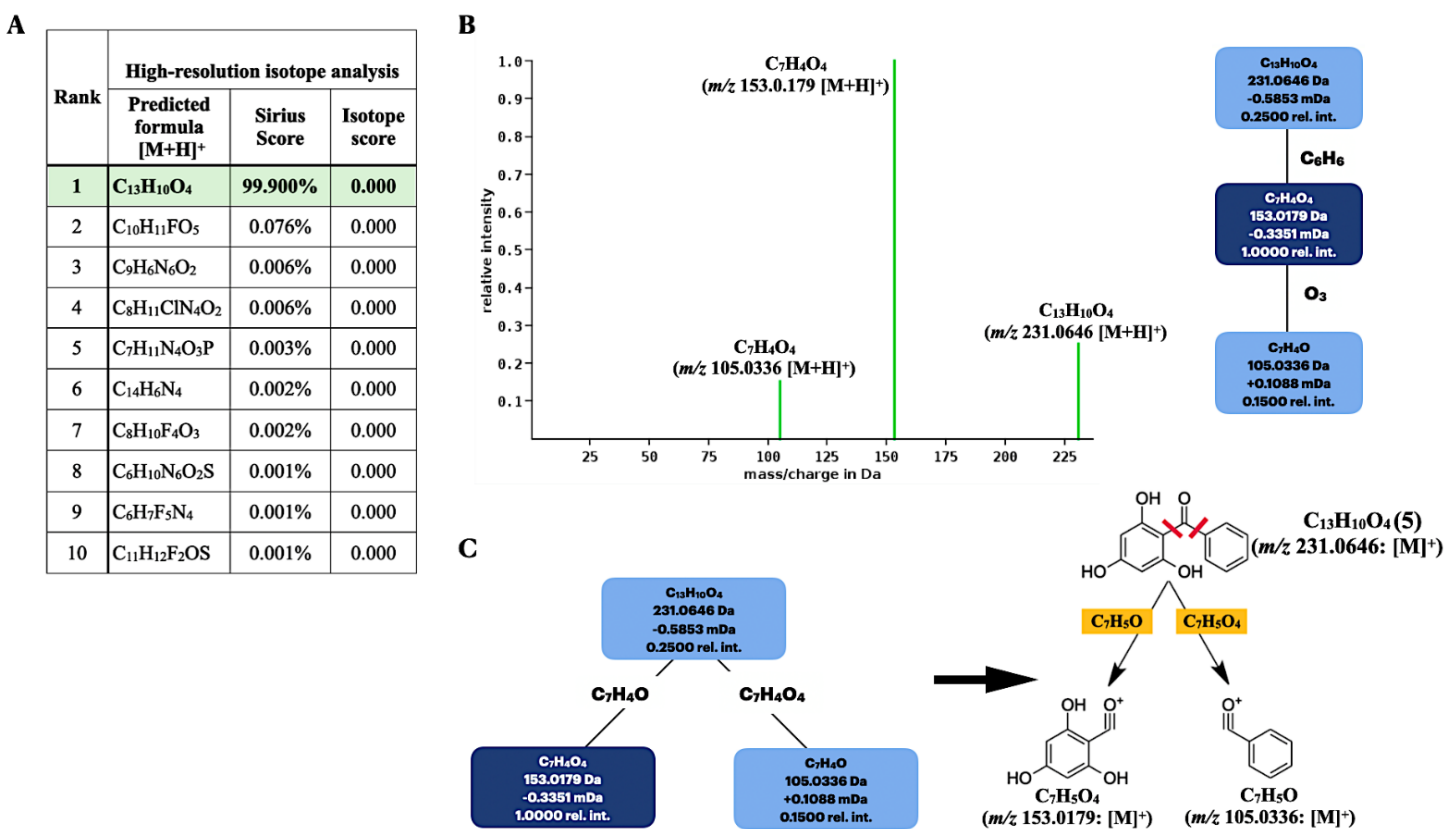
Molecular formula and fragmentation tree annotations of the query metabolite (*m*/*z* 231.0646 [M + H]^+^) produced by the clones carrying pETDuet-BadA-GmBPS by using the Sirius tool. (**A**) Based on a high-resolution isotope pattern analysis, the molecular formula of the query subject was best explained as C_13_H_10_O_4_. (**B**) The fragmentation tree simulated by the Sirius tool. (**C**) The proposed fragmentation pathway based on the alpha-cleavage sites of ketones to give two daughter ions with *m*/*z* 153.018 and 105.033.

Among the ten elemental formulas retrieved, the molecular formula of the secreted product (*m*/*z* 231.0646 [M + H]^+^) was best annotated as C_13_H_10_O_4_ with the highest prediction score, 99.900% (Figure 5A). This result corresponded to the standard 2,4,6-TriHB (**5**) reported in the PubChem database showing the molecular formula C_13_H_10_O_4_ (Figure 5A), suggesting they were the same substance. Sirius also simulated the fragmentation tree to provide the correlation between molecular ion *m*/*z* 231.0646 and the two fragmented ions (153.0179 and 105.0335) (Figure 5B). It was suggested that the molecular ion with *m*/*z* 231.0646 (C_13_H_10_O_4_) underwent the consecutive losses of C_6_H_6_ and O_3_ and yielded the two relevant product ions, with *m*/*z* 153.0180 (C7H4O4) and 105.033 (C7H4O), respectively. The suggested fragmentation pattern seemed to be unusual, especially the O_3_ loss, as it was distinguished from the typical fragmentation pattern of ketones where the two carbons adjacent to the carbonyl group undergo alpha-cleavages and McLafferty rearrangements [56]. Although this did not affect the overall molecular formula identification of the query subject, we suggested that the two alpha carbons located in 2,4,6-TriHB (**5**) were well suited for the alpha-cleavages to yield the two fragmented ions (*m*/*z* 153.180 and 105.0333), rather than those predicted by the Sirius tool. The proposed fragmentation pathway is also shown in Figure 5C.

**Figure 6:**
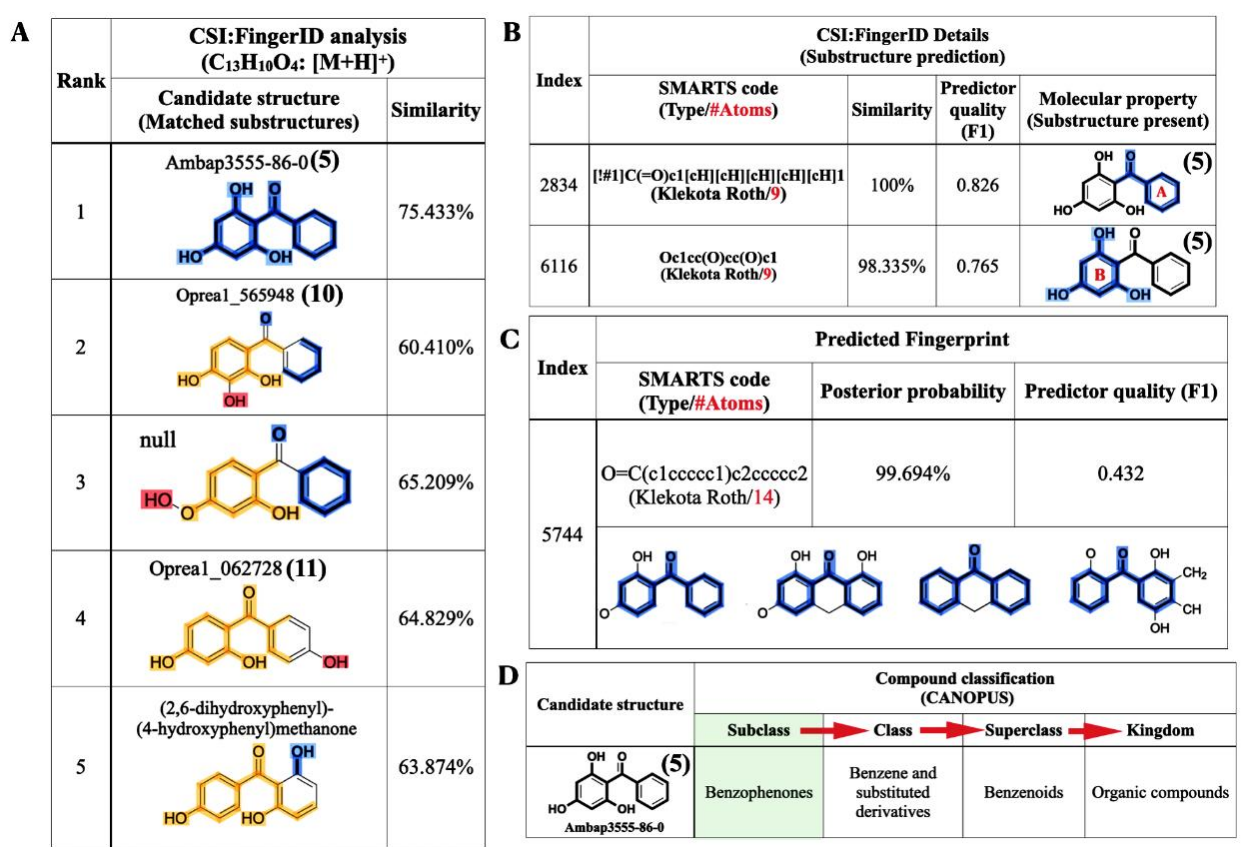
Structural annotations of the query metabolite (C_13_H_10_O_4_: *m*/*z* 231.0646 [M + H]^+^) produced by clones harboring pETDuet-BadA-GmBPS, as found using the CSI:FingerID web service and the CANOPUS tool. (**A**) CSI:FingerID revealed that the query metabolite was perfectly matched with 2,4,6-TriHB (**5**) (named Ambap3555-86-0). (**B**) The example substructures that corresponded to the A and B ring systems of 2,4,6-TriHB (**5**) predicted to be present in the query subject. (**C**) The basic benzophenone skeleton (BBS), one the molecular properties had predicted to be present in the training data of CSI:FingerID. (**D**) CANOPUS showing the query compound that belonged to the benzophenones, which explained the organic compounds containing two phenyl groups attached via a ketone bridge. The present substructures in the query metabolite were highlighted as intense blue lines.

As mentioned, SIRIUS was also connected with the CSI:FingerID web service to identify the compound structure, based on the experimental mass spectra of the query metabolites. Of the 100 candidate structures retrieved, our query subject (*m*/*z* 231.0646) was best annotated with 2,4,6-TriHB (**5**) (namely, Ambap3555-86-0), having the highest similarity score, 75.407%, with it (Figure 6A). CSI:FingerID also provides the so-called “molecular fingerprints” that help confirm the substructures present in the candidate structures. In this case, several substructures belonging to 2,4,6-TriHB (**5**) were predicted to be present in the query metabolite (C_13_H_10_O_4_: *m*/*z* 231.0646). For instance, the substructure encoded as a SMARTS string “[!#1]C(=O)c1[cH][cH][cH][cH][cH]1” scored 100% (F1 = 0.826), corresponding to the A-ring system of 2,4,6-TriHB (**5**) (Figure 6B). This chemical moiety originated from the benzoate (**2**) supplied in the culture of the engineered strain carrying pETDuet-BadA-GmBPS. CSI:FingerID also found phloroglucinol (1,3,5-trihydroxybenzene) in the same candidate structure, encoded as the SMARTS string “Oc1cc(O)cc(O)c1”, which possessed 98% similarity and achieved an F1-score of 0.765 (Figure 6B). This moiety served as the B-ring system of 2,4,6-TriHB (**5**), originating from the successive cyclization of the three units of malonyl-CoA (**4**) via the catalytic activity of GmBPS. The basic benzophenone skeleton (BBS) was predicted to be present in the trained structures with a posterior probability of 99.694% (F1 = 0.432) (Figure 6C). Based on the fingerprints predicted by CSI:Finger ID, CANOPUS subsequently confirmed that our query metabolite was classified as a benzophenone, for which benzene-substituted derivatives, benzenoids, and organic compounds were their molecular ancestors (Figure 6D).

### 2.4. Molecular Formula and Structural Annotation from the Negative Mode Mass Data

**Figure 7:**
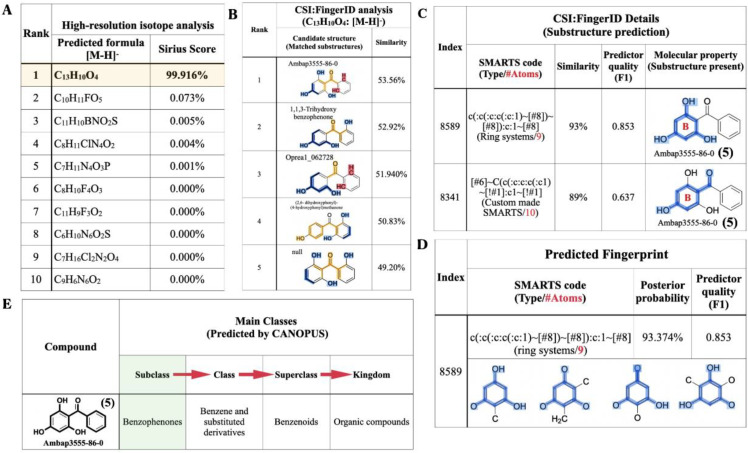
The molecular formula and structural annotation of the query metabolite (*m*/*z* 229.0496 [M − H]^−^), which was produced by clones carrying pETDuet-BadA-GmBPS. (**A**) A high-resolution isotope pattern analysis clearly showed that the molecular formula of the query was well annotated as C_13_H_10_O_4_ (99.916%); (**B**) The CSI:FingerID web service revealed that the compound (C_13_H_10_O_4_) was elucidated as 2,4,6-TriHB (**5**) (named Ambap3555-86-0). (**C**) Phloroglucinol (1,3,5-trihydroxybenzene) and the B-ring attached to the carbonyl group were the substructures predicted to be present in the query metabolite. (**D**) The substructures corresponding to phloroglucinol were detected in the CSI:FingerID training set. (**E**) CANOPUS, the compound classification tool, reveals that our query subject belonged to the class benzophenones, which are defined as the organic compounds containing two phenyl groups attached via a ketone bridge. The present substructures in the query metabolite were highlighted as intense blue lines.

The raw mass data of 2,4,6-TriHB (**5**) showing *m*/*z* 229.0496 [M − H]^−^ was also annotated by SIRIUS to obtain the most accurate and reliable prediction. Based on a high-resolution isotopic analysis, the molecular formula of the query subject was elucidated as C_13_H_10_O_4_ (99.916%), which was consistent with the exact formula of 2,4,6-TriHB (**5**) reported in several databases (Figure 7A). CSI:FingerID revealed that our query subject was best annotated as 2,4,6-TriHB (**5**) over the 100 molecular-structure candidates (Figure 7B). Several substructures belonging to 2,4,6-TriHB (**5**) were detected to be present, for example, the molecular fingerprint encoded by “c(:c(:c:c(:c:1)~[#8])~[#8]):c:1~[#8]” represented the B-ring system of 2,4,6-TriHB (**5**) (Figure 7C). The substructure corresponding to the B-ring system connected to the carbonyl group of benzoate (**2**) was also predicted to exist in the same candidate structure. CSI:FingerID showed the presence of molecular property corresponding to phloroglucinol in the trained structures with the posterior probability of 93.374% (F1 = 0.853) (Figure 7D). CANOPUS revealed that our query compound was classified as a benzophenone, and that benzene derivatives and benzenoids served as its molecular class ancestors (Figure 7E).

The obtained evidence indicated that the SIRIUS tool could strengthen the information regarding the molecular formula, substructures, and molecular structure of the query 2,4,6-TriHB (**5**), which unraveled from the experimental MS/MS spectra. Based on gathering information from both sides—the experiments and in silico predictions—it was obvious that the bioconversion of benzoate (**2**) by the clones carrying pETDuet-BadA-GmBPS led to the formation of 2,4,6-TriHB (**5**), rather than other isomeric structures of benzophenones, such as 2,3,4-TriHB (**10**) and 2,4,4-TriHB (**11**).

## 3. Discussion

2,4,6-TriHB (**5**) serves as the BBS of biologically active benzophenones and xanthones, which are exclusively found in plants in Clusiaceae and Hypericaceae [6,57]. Although the genes encoding for BPSs have been cloned from many plants [41,42,43,44], an attempt to establish a bacterial system for the production of this compound has not been reported, thus far. As a proof-of-concept experiment, we demonstrated that the *E. coli* BL21(DE3)pLysS strain could be engineered to produce 2,4,6-TriHB (**5**) from fed benzoate (**2**) through the coexpression of BadA and GmBPS. Our results proved and supported the previous findings that many plant polyketides (e.g., stilbenoids, flavonoids, and curcuminoids) can be produced by engineered *E. coli* fed with the low-priced precursors, such as 4-coumaric acid, caffeic acid, and ferulic acid [58,59,60,61]. 

As demonstrated, 2,4,6-TriHB (**5**) could be detected in both the pellets and the culture medium of clones bearing pETDuet-BadA-GmBPS (Figure 4C,D). This suggested that this compound was intracellularly synthesized in bacterial cells and transported into the medium, which is typical for the production of plant-specific polyketides by engineered *E. coli* [37,38,39]. From an economic point-of-view, the extracellular secretion of compounds into the culture medium provides a great advantage for downstream processes, as no breaking-cells step is required. The extracellular secretion of 2,4,6-TriHB (**5**) might be preceded by the membrane transporter proteins of *E. coli* BL21(DE3)pLysS, which attempted to alleviate the cellular toxicity from the accumulation of artificial compounds [62]. It has been demonstrated that the *E. coli* BL21 strain exported the PKS’ products (e.g., resveratrol, naringenin, and rutin) into the culture media by using the membrane transporter proteins, included the outer membrane protein A precursor (OmpA) and long-chain fatty acid transport protein (Fadl) [62]. The overexpression of the aromatic amino acid exporter (YddG), arabinose-proton symporter (AraE), outer membrane protein W (OmpW), and outer membrane protein F (OmpF) in the engineered *E. coli* BL21(DE3) strain resulted in the improved secretion of resveratrol into the culture medium [63]. Thus, upregulation of those transporter proteins might be beneficial for the improved secretion of 2,4,6-TriHB in the future. 

Benzoic acid, a protonated form of benzoate (**2**), (characterized by *m*/*z* 123.0442 [M + H]^+^) was detected in both the pellets and the culture medium of clones harboring pETDuet-BadA-GmBPS, signifying the incomplete conversion of the substrate (Figure S2). This result also indicated that the fed benzoate (**2**) was able to penetrate inside the bacterial cells but was incompletely converted into 2,4,6-TriHB (**5**), which might be due to the incomplete function of BadA or the loss of benzoyl CoA (**3**) through the catalytic function of the native *E. coli* acyl CoA thioesterases, such as EntH (YbdB), which catalyzes benzoyl-CoA (**3**) into benzoic acid [64,65]. It has been reported that the inhibition of 1,4-dihydroxy-2-napthoyl-CoA hydrolase (YdiL) catalyzes the conversion of salicoyl-CoA into salicylate and leads to a drastic increase in the production of 4-hydroxycoumarin (~300%) in metabolically engineered *E. coli* [66]. Thus, preventing benzoyl-CoA (**3**) loss via the inhibition of certain acyl-CoA thioesterases (i.e., YbdB) might also be a promising target in the metabolic engineering of 2,4,6-TriHB (**5**) reported herein.

The low bioconversion of benzoate might also result from the optimum pH of BadA, which prefers pH > 8.5 [47,48], rather than the cellular pH of *E. coli*, which is in the range of 7.2 to 7.8 [67,68]. Therefore, the replacement of the BadA gene with the other acyl-CoA ligases capable of catalyzing the formation of benzoyl-CoA (**3**) from benzoate (**2**) under the physiological pH of the host strains might be beneficial for the titer/yield of 2,4,6-TriHB (**5**). A previous study demonstrating the formation of benzoyl anthranilates in *S. cerevisiae* based upon the coexpression of BadA with hydroxycinnamoyl/benzoyl-CoA:anthranilate *N*-hydroxycinnamoyl/benzoyltransferase (HCBT) was not successful [69]. Those yields could be increased by changing BadA into 4-coumarate-CoA ligase (4CL5) from *Arabidopsis thaliana* [69].

Type III polyketide synthases (PKSs) typically generate small amounts of the derailment triketide lactone (**8**) and tetraketide lactone (**9**) that could not be fully expanded and/or were incorrectly folded into specific polyketides such as resveratrol, naringenin, and 2,4,6-TriHB (**5**) [41,70,71,72]. From a metabolic engineering point of view, the formation of these two ketide lactones indicated intermediate losses, which subsequently affected the yields of the final product. Herein, the two ketide lactones (**8** and **9**) were detected as minor products in the enzymatic reaction of GmBPS (Figure 3A), while they were absent in the culture medium of clones harboring pETDuet-BadA-GmBPS (Figure 3B). Our results were not consistent with previous findings that triketide and tetraketide lactones were derailment products of the metabolic engineering of flavonoids and stilbenoids in *E. coli* [58,72]. Based on the obtained evidence, however, it was still not clear whether the two derailment products produced by clones harboring pETDuet-BadA-GmBPS were below the detection limit of the LC-MS/MS systems used in this study or whether the tetraketide intermediate (Figure 1) catalyzed by the intracellular GmBPS underwent a smooth transition from the C6 → C1 Claisen condensation to specifically give 2,4,6-TriHB (**5**). Further experiments (e.g., upscaled production, optimized LC-MS condition) are required to gain insight into the de novo formation of 2,4,6-TriHB (**5**) and the two ketide lactones products in *E. coli*.

There are currently two methods designed for the production of 2,4,6-TriHB (**5**), total synthesis and enzymatic synthesis [4,41,42,43,44,45]. The first method relies on the use of phloroglucinol and benzonitrile as starting agents, which is quite expensive. Moreover, this method requires other substances, including ZnCl2, HCl, and dry ether, with multiple steps to obtain the 2,4,6-TriHB, a process that is costly and not friendly to the environment. Although the enzymatic synthesis of 2,4,6-TriHB (**5**) seems less complicated and more environmentally friendly than the total synthesis, this approach requires benzoyl-CoA (**3**) and malonyl-CoA (**4**), which are both high-priced substrates. Besides, multiple steps of protein expression, isolation, and purification to acquire the benzophenone synthase (BPSs) as an enzyme source to be used in this reaction are needed. Due to the low price of sodium benzoate, the use of this precursor for synthesizing 2,4,6-TriHB (**5**) via our engineered strain seems more appropriate and cost-effective. *E. coli* can also naturally supply many precursors needed for 2,4,6-TriHB (**5**) biosynthesis through its basal metabolisms, e.g., ATP, CoA, and malonyl-CoA (**4**), which reduces the chemicals consumed [37]. In doing this, 2,4,6-TriHB (**5**) could be produced in a short period of time (approximately 18 hours) and could be extracted from the *E. coli* culture medium by the appropriate downstream process.

Although NMR elucidation plays an important role in verifying the chemical structure of the target metabolites, this technique is limited to research into metabolic engineering and metabolomics, which is needed to explore the occurrence of the trace targeted metabolites [73,74,75,76]. The use of in silico tools to help with the structural annotation and elucidation of the target metabolites is possible in metabolic engineering, since the tools are typically used in the field of metabolomics. A recent study demonstrated that Sirius software could be used to validate the correct identification of two metabolites from 5-fluorouracilin metabolism, i.e., fluorouridine (FURD) and fluorodeoxyuridine (FdURD), in cancer-resistant cells, without using the reference standards [77]. 2,4,6-TriHB (**5**) was not used, and the yield of 2,4,6-TriHB (**5**) was extremely low. The confirmation of the existence of 2,4,6-TriHB produced by our engineered strain is further strengthened by using SIRIUS software integrated with CSI:FingerID and CANOPUS, resulting in the identification of various chemical features that indicated the bioconversion product is highly likely to be 2,4,6-TriHB (**5**), including the correct molecular formula (C_13_H_10_O_4_), the chemical structure predicted to be the same as 2,4,6-TriHB (**5**), and its classification as a benzophenone.

An artificial pathway of 2,4,6-TriHB (**5**) was successfully established in *E. coli*, however, the obtained results can only be regarded as an initial step in the field of synthetic biology because the production of the target product is still at a very low level. An improved 2,4,6-TriHB (**5**) yield is needed to serve large-scale applications. Those might include the insertion of acetyl-CoA carboxylases (ACCs) to elevate the malonyl-CoA (**4**) pool and the inhibition of the endogenous acyl-CoA thioesterase’s (e.g., YbdB) catalytic function to minimize benzoyl-CoA loss. 

Furthermore, based on the ring-B system of 2,4,6-TriHB (**5**) that contains electron-rich regions, structural modifications (i.e., prenylation) [78,79] through Friedel–Crafts aromatic electrophilic substitution would have a high potential. This suggests establishing the newly modified clones carrying pETDuet-BadA-GmBPS, coexpressed with prenyltransferases (PTs) and xanthone synthases (XSs) for the synthesis of prenylated benzophenones and xanthones, respectively.

## 4. Materials and Methods

### 4.1. Reagents

The general reagents were analytical grade and were purchased from Sigma-Adrich (St. Louis, MO, USA), Merck (Darmstadt, Germany), Himedia Laboratories (Munbai, India), and Avantor (Center Valley, PA, USA).

### 4.2. Enzymatic Preparation of 2,4,6-TriHB *(**5**)*

The preparation of 2,4,6-TriHB (**5**) was performed as described by Nualkaew et al. (2012) with some modifications [41]. The crude lysate (containing GmBPS protein) prepared from *E. coli* BL21(DE)pLysS-carried pET22b-GmBPS was purified using Histrap FF crude column 1 mL (GE Healthcare, Buckinghamshire, UK). Briefly, the crude protein was loaded onto the column, which was pre-equilibrated with a 20 mM sodium phosphate buffer with a pH of 7.4, containing 500 mM NaCl and 20 mM imidazole, then was washed with the same buffer and eluted with an elution buffer with pH 7.4 (20 mM sodium phosphate buffer pH 7.4 containing 500 mM NaCl and 500 mM imidazole). The recombinant protein was desalted, concentrated, and changed into 20 mM Tris/HCl with pH 8 using a Vivaspin centrifugal concentrator (MWCO 10 kDa cutoff, Vivascience AG, Hannover, Germany). The enzymatic reaction (500 μL) consisted of 108 μM benzoyl-CoA (**3**), 216 μM malonyl-CoA (**4**), 200 μg partially purified GmBPS, and a 100 mM potassium phosphate buffer, pH 7.0, containing 1 mM EDTA. The mixture was incubated with shaking at 300 rpm, 30 °C for 20 h, then terminated by adding 20% HCl (50 μL), followed by extraction with 500 μL EtOAc (3 times). The EtOAc layers were collected, dried under N2 gas and redissolved with 20 μL methanol (HPLC-grade) before being analyzed by a high-resolution LC-ESI-QTOF-MS/MS at the central instrument, Salaya, Nakhon Pathom, Thailand.

### 4.3. Construction of Plasmid pETDuet-BadA-GmBPS

The pETDuet-1 coexpression vector (Novagen, Germany) was used to create a new vector containing BadA and GmBPS (namely, pETDuet-BadA-GmBPS), based on the strategy described by Toila and Joshua-Tor (2006) [51]. In this study, BadA was inserted into the MCS-1 (*BamH*I&*Not*I), while the GmBPS was incorporated into the MCS-2 (*Nde*I&*Xho*I). Due to an open reading frame (ORF) of BadA that contained the *Xho*I recognition site (Figure S3), the initial incorporation of GmBPS into the MCS-2 of the pETDuet-1 vector, followed by the insertion of BadA into the MCS-1, was performed instead. The strategy was as follows: The plasmid pET22b-GmBPS from Nualkaew et al., 2012 [41], was the DNA template for the amplification of gene-encoded GmBPS (~1176 bp). The PCR reaction contained Ex-Taq DNA polymerase (Takara, Shiga, Japan); the forward primer was GmBPS-F 5′-TGCCATATGGCACCTGCAATGGATTCT-3′, and the reverse primer was GmBPS-R 5′-AGCCTCGAGTGCTATTGGCACACTACG-3′ (underlines are the restriction sites for *Nde*I and *Xho*I, respectively). The PCR cycle comprised predenaturation at 98 °C for 30 s, followed by 30 cycles of 98 °C for 30 s, 55 °C for 30 s, and 72 °C for 1 min, followed by a final extension at 72 °C for 5 min. The PCR product (~1176 bp) was purified using a Gel Band Purification Kit (GE Healthcare, Chicago, IL, USA), double-digested with *Nde*I and *Xho*I, and ligated into a pETDuet-1 vector that had been treated with the same restriction enzymes. The ligation mixture was transformed into *E. coli* DH5α and spread on a Luria–Bertani (LB) agar plate containing ampicillin (100 μg/mL). The positive clones carrying pETDuet-GmBPS were selected by colony PCR based on the gene-specific primer for GmBPS (GmBPS-F and GmBPS-R). The resulting plasmids were extracted using the PureYieldTM Plasmid Miniprep System (Promega, Madison, WI, USA) and used as the DNA backbone in the next step. 

**Figure 8:**
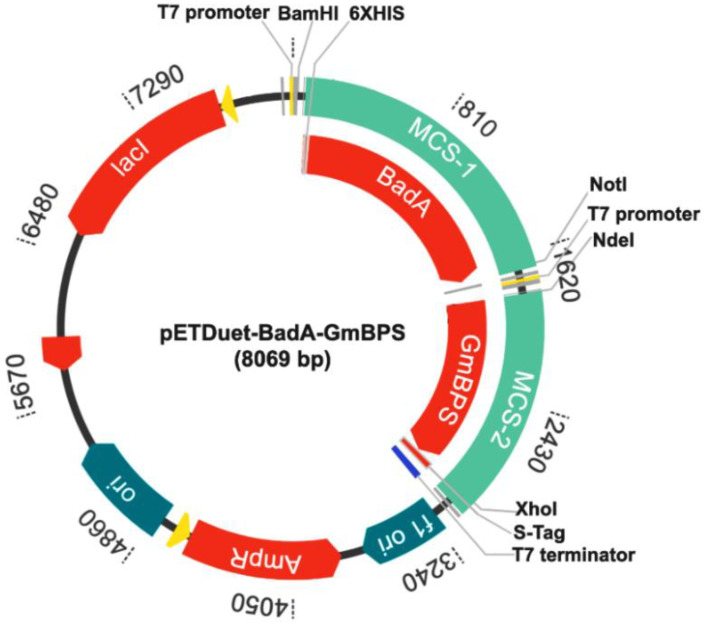
The recombinant plasmid pETDuet-BadA-GmBPS.

The pMK-RQ-BadA was synthesized by GeneArt Gene Synthesis (Thermo Fisher Scientific, Waltham, MA, USA) and used as the DNA template for the amplification of BadA (1566 bp). The PCR reaction consisted of Phusion High-Fidelity DNA Polymerase (Thermo Fisher Scientific, Waltham, MA, USA), BadA-F: 5′-GCGGGATCCTATGAATGCAGCCGCGGT-3′, and BadA-R: 5′-AATGCGGCCGCTTCAGCCCAACACACCCTC-3′ (underlines are the restriction sites for *BamH*I and *Not*I, respectively). The PCR condition was as follows: predenaturation at 98 °C for 1 min, followed by 30 cycles of 98 °C for 30 s, 60 °C for 30 s, 72 °C for 30 min, and a final extension at 72 °C for 5 min. The PCR product was purified using a gel purification kit (GE Healthcare, USA), doubly digested with *BamH*I and *Not*I, and ligated with the pETDuet-GmBPS that had been cut with the same restriction enzymes. The ligation mixture was transformed into *E. coli* DH5α and selected to obtain positive clones by spreading it on a Luria–Bertani (LB) agar plate containing ampicillin (100 μg/mL). The resulting plasmids were extracted using the PureYieldTM Plasmid Miniprep System (Promega, USA). Gene insertion was confirmed by *BamH*I digestion, based on monitoring the cut DNA exhibiting a size of 8069 bp (pETDuet-1+BadA+GmBPS). Nucleotide sequencing was performed to verify the correct bases and the in-frame arrangement of BadA and GmBPS using two pairs of primers: pETUpstream primer 5′-ATGGCTCCGGCGTAGA-3′ and DuetDOWN1 primer 5′-GATTATGCGGCCGTGTACAA-3′ for BadA in the MCS-1, and DuetUP2 primer 5′-TTGTACACGGCCGCATAATC-3′ and T7term primer 5′-GCTAGTTATTGCTCAGCGG-3′ for GmBPS in the MCS-2. DNA sequencing showed that both genes were inserted into the expected regions of the pETDuet-1 vector. The BadA gene was placed downstream of the transcription-controlling regions, including the T7 promoter-1, lac operator, and ribosome binding site (rbs) of the vector (Figure S4). The His-tag sequence was linked at the 5′-terminal of this gene. The GmBPS gene was inserted beside the T7 promoter-2, lac operator, and rbs in the MCS-2 (Figure S5) and was fused with S.Tag at the C-terminal. The map of pETDuet-BadA-GmBPS (8069 bp), created by using GenScript (https://www.genscript.com/gensmart-design/# (accessed on 3 March 2021, is shown in Figure 8.

### 4.4. Bioconversion of Benzoate *(**1**)* to 2,4,6-TriHB *(**5**)*

pETDuet-BadA-GmBPS (8069 bp) was transformed into *E. coli* BL21(DE3) pLysS (Promega, USA) by using the heat-shock method according to manufacturer recommendations. The clones carrying pETDuet-BadA-GmBPS were cultured in the LB medium containing ampicillin (100 μg/mL) and chloramphenicol (34 μg/mL) at 37 °C and 200 rpm for 18 h. The 1.5 mL of fresh culture was inoculated into the 500 mL Erlenmeyer flask containing 150 mL of the same medium and cultivated at 37 °C (200 rpm) until the OD_600_ reached 0.8–1.0. The heterologous expression of BadA and GmBPS was induced by supplementing IPTG (1 mM). The cells were grown at 18 °C (250 rpm) for 5 h before adding 5 mM sodium benzoate (**1**) and 3 mM MgCl_2_ to start bioconversion, then the cells were further cultured at the same conditions for 18 h. The cell pellets and culture medium were harvested by centrifugation at 4 °C and 8000 rpm for 10 min. The *E. coli* BL21(DE)pLysS-harbored pETDuet-1 empty vector grown in parallel at the same culture condition was the control in this study.

### 4.5. Metabolite Extraction from the Cell Pellets

The obtained cell pellets were washed twice in sterile water to minimize contamination from the residues of the culture medium. Then, the pellets were initially disrupted by using freeze-thaw at −80 °C and 37 °C, followed by incubation in 20 mL of lysis buffer with pH 7.4, containing 20 mM sodium phosphate, 1% tween-20, and 1 mg/mL lysozyme in the ice bath for 1.5 h. The clear lysate was harvested by centrifugation at 4 °C (10,000 rpm) for 30 min. The obtained clear lysate was partitioned twice with an equal volume of EtOAc in a 250 mL Erlenmeyer flask at 25 °C and 300 rpm for 30 min, then centrifuged at 6000 rpm at 4 °C for 5 min to separate the organic layer. The EtOAc layer was transferred to a new tube and evaporated until dry under a gentle stream of N_2_ gas. The residues were dissolved in 150 μL of methanol (HPLC grade) before the detection of the intracellular 2,4,6-TriHB (**5**) and associated metabolites by using high-resolution LC-ESI-QTOF-MS/MS.

### 4.6. Extraction of the Secreted Metabolites from the Culture Medium 

The culture medium (150 mL) was extracted twice with 75 mL of EtOAc in a 500 mL Erlenmeyer flask by shaking at 300 rpm and 25 °C for 30 min. The EtOAc layers were separated by centrifugation for 5 min and 6000 rpm at 4 °C, then transferred to the new tube and dried under a gentle stream of N_2_ gas. The dried residues were dissolved in 150 μL of methanol (HPLC grade). The secretion of 2,4,6-TriHB (**5**) and related metabolites were measured by using the high-resolution LC-ESI-QTOF-MS/MS. 

### 4.7. Metabolite Detection by Using the High-Resolution LC-ESI-QTOF-MS/MS

The identification of 2,4,6-TriHB (**5**) and associated metabolites was carried out by using an HPLC Ultimate 3000 RSLC (Thermo Fisher Scientific, Bremen, Germany) connected with a mass spectrometer, Maxis (Bruker Daltonics, Bremen, Germany), equipped with the Acclaim RSLC 120 C18 column (2.1 × 100 mm^2^, 2.2 μM) (Thermo Fisher Scientific, Waltham, MA, USA). The mobile phases consisted of water with 0.1% formic acid (solvent A) and acetonitrile with 0.1% formic acid (solvent B). The elution was 0.4 mL/min using a linear gradient of solvent B as follows: 5% for 0–2 min, 5–95% for 15 min, 95% for 3 min, and back to 5% for 5 min, for a total runtime of 25 min. The sampler temperature was set to 10 °C. The column oven temperature was 40 °C. The injection volume was 5 μL. The nebulizer pressure was set at 29 psi; the dry gas temperature was 180 °C, and the dry gas flow rate was 8.0 L/min. Product identification was then performed using tandem mass spectrometry (MS/MS) with electrospray ionization (ESI) coupled with multiple reaction monitoring (MRM). The collision-induced dissociation (CID) energy was 20 eV in positive ion mode analysis, where the masses were scanned over the *m*/*z* range of 100–1000 amu. The samples were also analyzed under the same chromatographic separation condition except that the ion polarity was switched to the negative ion mode detection with the CID of 35 eV (mass scan range 50–1500 amu). 

The total ion chromatogram (TIC) and MS/MS spectra of 2,4,6-TriHB (**5**) acquired from the bioconversion of benzoate (**1**) by clones carrying pETDuet-BadA-GmBPS were identified by comparing them against the fragmentation pattern of standard 2,4,6-TriHB (**5**) prepared from the in vitro reaction of GmBPS and those deposited in the PubChem database in both positive and negative ion ionization modes. The putative mass spectra of triketide lactone (**8**) and tetra ketide lactone (**9**), the derailment products of GmBPS, were identified with those established by our group (Figures S6 and S7) [41]. The raw mass data of putative 2,4,6-TriHB (**5**) (Figures S8 and S9) were further annotated by the SIRIUS tool (version 4.5) to acquire more detailed information about the molecular formula, chemical structure (couple with the relevant substructures), and compound classifications of the query subject to verify the correction of the obtained results.

### 4.8. Identification of 2,4,6-TriHB *(**5**)* Using an in Silico Mass Prediction Tool

It is generally accepted that NMR elucidation plays a crucial role in confirming the chemical structures of the target metabolites; consequently, the use of this technique is limited to preliminary studies, especially for metabolic engineering and metabolomics, in which the yields of final products are typically low [70,71,72,73]. SIRIUS is one of the computational tools recently designed for identifying the correct molecular formula of query metabolites based on a high-resolution isotopic pattern analysis for fulfilling this bottleneck [51,52]. For efficient structural elucidation, this tool is also integrated with others, including the CSI:FingerID web service, fragmentation tree, and CANOPUS, to provide vital information regarding the chemical structure, substructures, parent-product ion correlations, and compound classes [53,54,55]. This tool has recently been used in the metabolomic studies of 5-fluorouracil metabolisms in cancer cells with acquired chemoresistance [76]. Therefore, SIRIUS (version 4.5) was used for the structural elucidation of putative 2,4,6-TriHB (**5**), based on the raw mass data run in both positive and negative modes. The analysis was conducted according to the user manual. Briefly, the raw MS/MS data in text format was imported directly into the SIRIUS application window. The MS2 level and collision energy were then defined in the subsequent dialogue. After that, two parameters, including the precursor mass ion and the adduct type, were defined in the following application window. After selecting the “compute option”, the annotation was implemented by the selection of SIRIUS, CSI:FingerID, and CANOPUS to identify the correct molecular formula, molecular structure, and compound categories, respectively. 

## 5. Conclusions

The *E. coli* BL21(DE3)pLysS strain was established as a biotransformation system for producing 2,4,6-TriHB (**5**) through the coexpression of BadA and GmBPS. Feeding sodium benzoate (**1**) into the culture of the engineered strain resulted in the in vivo formation of 2,4,6-TriHB (**5**), which was mainly secreted into the culture medium rather than accumulating in the cell pellets. The use of in silico tools including SIRIUS, CSI:FingerID, and CANOPUS led to the unraveling of the unique chemical features that were hidden in the raw MS/MS data, including the correct molecular formula, chemical structure, and compound classes, and clearly confirmed that the bioconversion product was indeed 2,4,6-TriHB (**5**). Further experiments might be needed to overcome the limited yield. The results suggested the potential use of this new strain of recombinant *E. coli* that utilizes sodium benzoate (**1**) as a feeding precursor for the further synthesis of prenylated benzophenones and xanthones.

## Data Availability

Not applicable.

## Supplementary Materials

Figure S1: the MS/MS spectra of 2,4,6-TriHB(-) reported in PubChem database; Figure S2: the mass spectrum of benzoic acid detected from the pellets (A) and culture medium (B) of clones carried pETDuet-BadA-GmBPS; Figure S3: the XhoI recognition site* detected in the BadA coding sequence; Figure S4: the insertion of BadA in the MCS-1 region of pETDuet-1 vector; Figure S5: the insertion of GmBPS into the MCS-2 region of pETDuet-1 vector; Figure S6: the MS/MS spectra of triketide lactone; Figure S7: the MS/MS spectra of triketide lactone; Figure S8: the raw mass data of 2,4,6-TriHB (**5**) measured by positive ion mode LC-MS/MS; Figure S9: the raw mass data of 2,4,6-TriHB (**5**) measured by negative ion mode LC-MS/MS.
