# Supplementary material for: Biotransformation of Benzoate to 2,4,6-Trihydroxybenzophenone by Engineered Escherichia coli
Source: Molecules. 2021 May 8;26(9):2779. doi: 10.3390/molecules26092779 (PMC8125937; doi:10.3390/molecules26092779)
Supplement: Supplementary file 1 [file molecules-26-02779-s001.zip › molecules-1189528-supplementary.pdf]

Supplementary Materials

Biotransformation of Benzoate to 2,4,6-Trihydroxybenzophenone by Engineered *Escherichia coli*

Anuwatchakij Klamrak [1], Jaran Nabnueangsap [2] and Natsajee Nualkaew [1]

[1] Faculty of Pharmaceutical Sciences, Khon Kaen University, Khon Kaen 40002, Thailand; anuwat_kla@yahoo.com (A.K); nnatsa@kku.ac.th (N.N)
[2] Salaya Central Instrument Facility RSPG, Mahidol University, Nakhon Pathom 73170, Thailand; jaran.nab@mahidol.ac.th

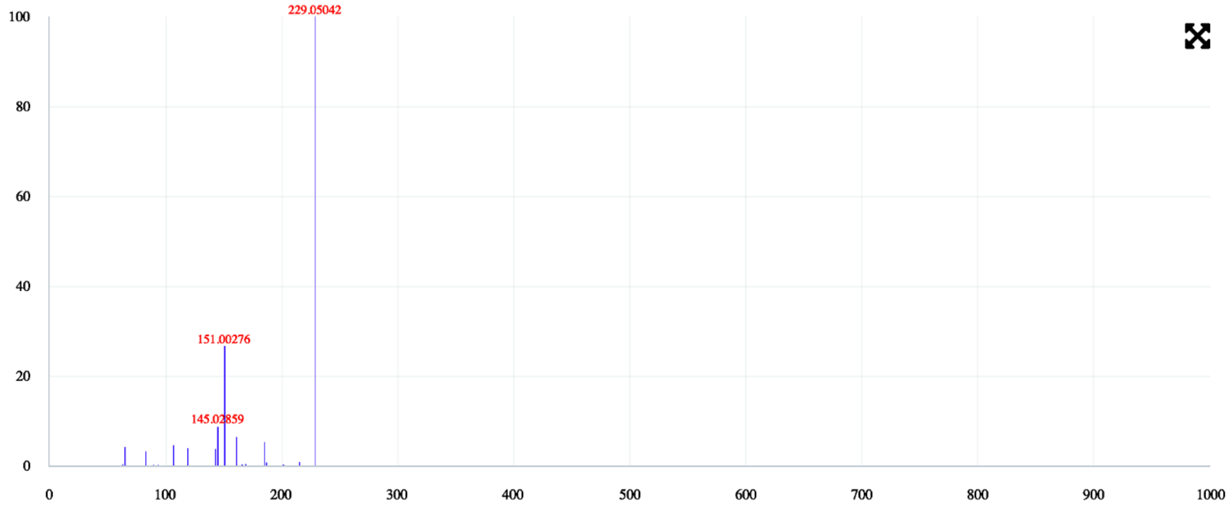

Figure S1. The MS/MS spectra of 2,4,6-TriHB(−) reported in PubChem database.

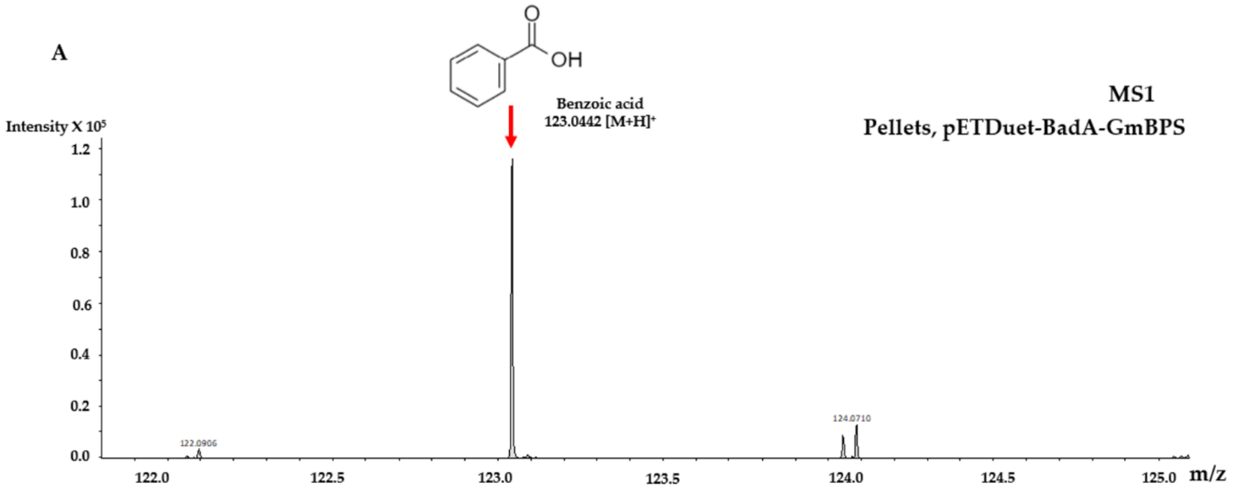

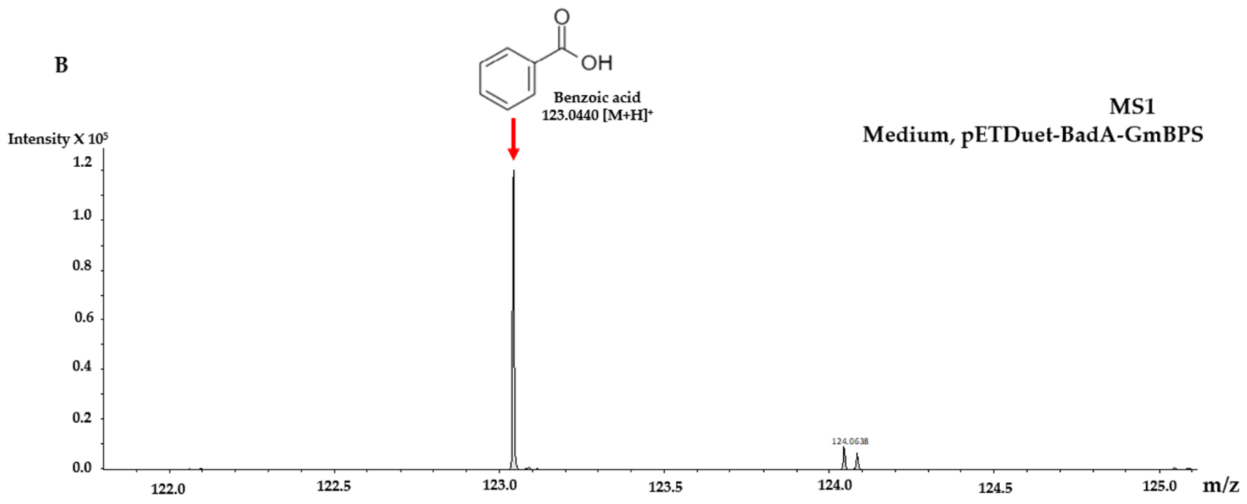

Figure S2. The EICs for the putative benzoic acid (calc. 123.044605 $[M+H]^+$) detected both from pellets (**A**) and medium (**B**) of clones harbored pETDuet-BadA-GmBPS.

>L42322.1:19-1584 Rhodopseudomonas palustris benzoate-coenzyme A ligase (badA) gene, complete cds

ATGAATGCAGCCGCGGTCACGCCGCCACCCGAGAAGTTTAATTTTGCCGAGCACCTGCTGCAGACCAATC

GCGTGCGGCCGGACAAGACGGCGTTCGTCGACGACATCTCGTCGCTGAGCTTCGCGCAACTCGAAGCTCA

GACGCGTCAGCTCGCCGCCGCCTTACGCGCGATCGGGGTGAAACGCGAAGAGCGCGTGCTGCTGCTGATG

CTCGACGGCACGGATTGGCCGGTGGCGTTTCTCGGCGCAATCTACGCCGGCATCGTGCCGGTCGCGGTCA

*Xho*I

ATACGCTGCTGACGGCGGACGACTACGCCTACATG**CTCGAG**CATTCGCGGGCTCAGGCCGTGCTGGTCAG

CGGCGCGCTGCACCCGGTGCTCAAGGCAGCGCTGACCAAGAGCGATCACGAGGTGCAGCGAGTGATCGTT

TCGCGCCCAGCGCTCCGCTGGAGCCGGGCGAGGTCGACTTCGCTGAGTCGGCGCACATCGCTTGAGAAGC

CTGCCGCTACGCAAGCGGACGATCCGGCGTTCTGGCTGTATTCGTCGGGTTCTACCGGGCGGCCGAAGGG

CGTGGTGCACACTCACGCCAATCCGTACTGGACCTCGGAGCTGTACGGCCGCAACACGCTGCATCTGCGC

GAAGACGACGTCTGCTTTTCGGCGGCCAAACTGTTTTTCGCTTACGGCCTCGGCAACGCGCTGACGTTTC

CGATGACGGTCGGCGCGACCACGCTGCTGATGGGCGAGCGACCGACGCCGGACGCGGTGTTCAAGCGCTG

GCTCGGCGGCGTCGGCGGTGTGAAACCGACCGTGTTCTACGGCGCGCCCACCGGCTACGCCGGCATGTTG

GCCGCGCCGAACCTGCCGTCGCGCGACCAGGTGGCGTTGCGGCTCGCGTCGTCGGCGGGCGAAGCACTGC

CGGCGGAGATTGGGCAGCGCTTCCAGCGCCATTTCGGCCTCGACATCGTCGATGGCATCGGCTCGACCGA

GATGCTGCACATCTTTCTGTCGAACCTGCCAGACCGGGTGCGCTACGGCACCACCGGATGGCCGGTGCCG

GGCTATCAGATCGAGCTGCGCGGCGACGGCGGCGGACCGGTCGCCGACGGAGAGCCGGGCGATCTCTACA

TTCACGGCCCGTCATCGGCGACGATGTATTGGGGCAACCGGGCCAAGAGCCGCGACACCTTCCAGGGCGG

CTGGACCAAGAGCGGCGACAAATACGTCCGCAACGACGACGGCTCCTACACCTATGCGGGCCGCACCGAC

GACATGCTGAAGGTCAGCGGCATCTATGTCAGCCCGTTCGAGATCGAAGCGACGCTGGTGCAGCATCCCG

GTGTGCTCGAAGCCGCAGTGGTCGGGGTCGCCGACGAACACGGCCTGACCAAACCGAAGGCCTATGTGGT

GCCGCGGCCCGGCCAGACCCTGTCGGAGACCGAGCTGAAGACCTTCATCAAGGATCGACTGGCGCCGTAC

AAATATCCGCGCAGCACGGTGTTCGTCGCCGAATTGCCGAAGACGGCGACCGGCAAGATTCAGCGCTTCA

AGCTGCGCGAGGGTGTGTTGGGCTGA

Figure S3. The *XhoI* recognition site* detected in the BadA coding sequence. * The restriction site was analysed by NEBcutter V2.0 (http://nc2.neb.com/NEBcutter2/).

GGGGAATTGATCGCGAATTAATACGACTCACTATAGGGGAATTGTGAGCGGATAACAATTCCCCTCTAGAAATAATT

RBS **6XHIS** ***Bam*HI** **Start codon**

TTGTTTAACTTTAAG**AAGGAG**ATATACCATGGGCAGCAGC**CATCACCATCATCACCAC**AGCCA**GGATCC**T**ATG**AATG

CAGCCGCGGTCACGCCGCCACCCGAGAAGTTTAATTTTGCCGAGCACCTGCTGCAGACCAATCGCGTGCGGCCGGAC

AAGACGGCGTTCGTCGACGACATCTCGTCGCTGAGCTTCGCGCAACTCGAAGCTCAGACGCGTCAGCTCGCCGCCGC

CTTACGCGCGATCGGGGTGAAACGCGAAGAGCGCGTGCTGCTGCTGATGCTCGACGGCACGGATTGGCCGGTGGCGT

TTCTCGGCGCAATCTACGCCGGCATCGTGCCGGTCGCGGTCAATACGCTGCTGACGGCGGACGACTACGCCTACATG

CTCGAGCATTCGCGGGCTCAGGCCGTGCTGGTCAGCGGCGCGCTGCACCCGGTGCTCAAGGCAGCGCTGACCAAGAG

CGATCACGAGGTGCAGCGAGTGATCGTTTCGCGCCCAGCGCTCCGCTGGAGCCGGGCGAGGTCGACTTCGCTGAGTC

GGCGCACATCGCTTGAGAAGCCTGCCGCTACGCAAGCGGACGATCCGGCGTTCTGGCTGTATTCGTCGGGTTCTACC

GGGCGGCCGAAGGGCGTGGTGCACACTCACGCCAATCCGTACTGGACCTCGGAGCTGTACGGCCGCAACACGCTGCA

TCTGCGCGAAGACGACGTCTGCTTTTCGGCGGCCAAACTGTTTTTCGCTTACGGCCTCGGCAACGCGCTGACGTTTC

CGATGACGGTCGGCGCGACCACGCTGCTGATGGGCGAGCGACCGACGCCGGACGCGGTGTTCAAGCGCTGGCTCGGC

GGCGTCGGCGGTGTGAAACCGACCGTGTTCTACGGCGCGCCCACCGGCTACGCCGGCATGTTGGCCGCGCCGAACCT

GCCGTCGCGCGACCAGGTGGCGTTGCGGCTCGCGTCGTCGGCGGGCGAAGCACTGCCGGCGGAGATTGGGCAGCGCT

TCCAGCGCCATTTCGGCCTCGACATCGTCGATGGCATCGGCTCGACCGAGATGCTGCACATCTTTCTGTCGAACCTG

CCAGACCGGGTGCGCTACGGCACCACCGGATGGCCGGTGCCGGGCTATCAGATCGAGCTGCGCGGCGACGGCGGCGG

ACCGGTCGCCGACGGAGAGCCGGGCGATCTCTACATTCACGGCCCGTCATCGGCGACGATGTATTGGGGCAACCGGG

CCAAGAGCCGCGACACCTTCCAGGGCGGCTGGACCAAGAGCGGCGACAAATACGTCCGCAACGACGACGGCTCCTAC

ACCTATGCGGGCCGCACCGACGACATGCTGAAGGTCAGCGGCATCTATGTCAGCCCGTTCGAGATCGAAGCGACGCT

GGTGCAGCATCCCGGTGTGCTCGAAGCCGCAGTGGTCGGGGTCGCCGACGAACACGGCCTGACCAAACCGAAGGCCT

ATGTGGTGCCGCGGCCCGGCCAGACCCTGTCGGAGACCGAGCTGAAGACCTTCATCAAGGATCGACTGGCGCCGTAC

AAATATCCGCGCAGCACGGTGTTCGTCGCCGAATTGCCGAAGACGGCGACCGGCAAGATTCAGCGCTTCAAGCTGCG

CGAGGGTGTGTTGGGC**TGA**GCGGCCGCATAATGCTAAGCGACAGAAATTGCCC

Stop codon ***Not*I**

Figure S4. The insertion of BadA in MCS-1 region of pETDuet-1 vector.

RBS

AGGGAAACTACTATAGGGGATTGTGAGCGGATAACAATTCCCCATCTTAGTATATTAGTTAAGTATAAG**AAGGAG**AT

Start codon

ATA**CATATG**GCACCTGCAATGGATTCTGCCCAAAATGGCCACCAAAGCCGAGGTTCTGCCAATGTTCTAGCAATTGG

***Nde*I**

CACTGCAAATCCACCAAACGTCATTCTGCAAGAAGACTATCCTGATTTTTACTTTAAAGTCACAAATAGCGAGCATT

TAACTGACTTGAAAGAAAAATTTAAGCGTATATGTGTTAAATCAAAAACCAGGAAGAGGCATTTTTATCTAACAGAG

CAAATCCTTAAAGAGAACCCAGGCATTGCAACCTATGGAGCAGGATCTCTCGACTCTCGTCAAAAGATTCTCGAAAC

GGAGATTCCTAAGCTCGGAAAAGAGGCTGCAATGGTAGCCATCCAAGAATGGGGACAACCAGTGTCGAAAATCACCC

ACGTTGTATTCGCCACCACTTCAGGCTTTATGATGCCCGGAGCTGACTACTCAATCACCAGGCTTCTCGGCCTAAAT

CCCAATGTAAGGCGCGTAATGATCTATAACCAAGGATGCTTTGCTGGTGGCACGGCCCTTCGTGTTGCCAAGGACCT

TGCAGAGAACAACAAGGGTGCTCGTGTCCTTGTAGTTTGCGCTGAGAACACAGCCATGACCTTCCACGGACCTAATG

AGAATCATTTAGATGTGTTGGTTGGTCAGGCTATGTTCTCTGATGGCGCGGCTGCTTTGATCATTGGAGCCAACCCC

AATTTACCAGAGGAACGTCCAGTTTATGAGATGGTAGCAGCCCATCAAACCATTGTCCCTGAGTCGGATGGGGCGAT

AGTTGCGCATTTTTATGAGATGGGAATGAGTTACTTCCTCAAAGAGAATGTTATTCCTCTTTTTGGCAACAACATTG

AGGCTTGCATGGAGGCCGCATTTAAGGAATATGGAATTAGTGACTGGAACTCCTTGTTCTATTCTGTGCATCCTGGT

GGCCGGGCTATCGTTGATGGAATCGCAGAGAAGTTGGGTCTTGATGAAGAAAACCTCAAGGCAACAAGGCACGTTTT

GAGTGAATATGGGAATATGGGTTCTGCTTGTGTCATCTTTATCCTCGATGAGCTGAGGAAGAAATCCAAAGAGGAGA

AGAAGCTCACCACTGGAGATGGAAAGGAGTGGGGTTGCCTTATTGGATTAGGCCCGGGACTCACCGTGGAGACCGTC

GTACTTCGTAGTGTGCCAATAGCA**CTCGAG**TCTGGT**AAAGAAACCGCTGCTGCGAAATTTGAACGCCAGCACATGGA**

***Xho*I** **S-Tag**

CTCGTCTACTAGCGCAGCTTTATTTCCTTTCCCCCT

Figure S5. The insertion of GmBPS in MCS-2 region of pETDuet-1 vector.

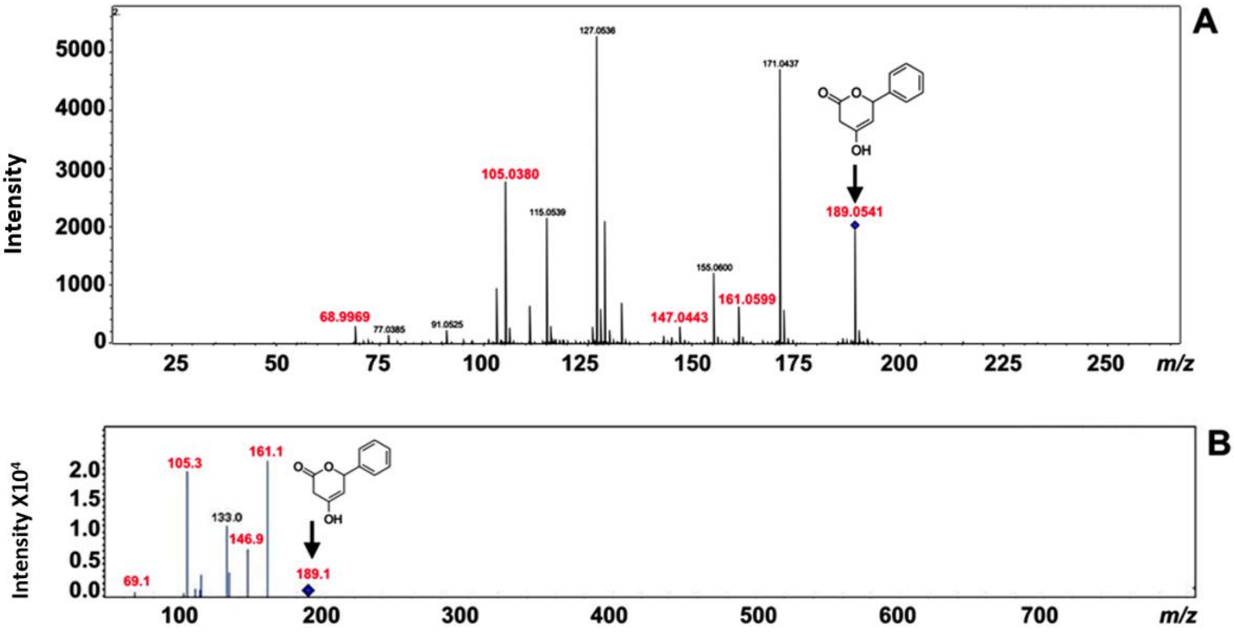

Figure S6. The MS/MS spectra of triketide lactone. (**A**) The MS/MS spectra of triketide lactone obtained from the enzymatic reaction of GmBPS reported in this study; (**B**) The MS/MS spectra of triketide lactone reported by Nualkaew et al. 2012 [12].

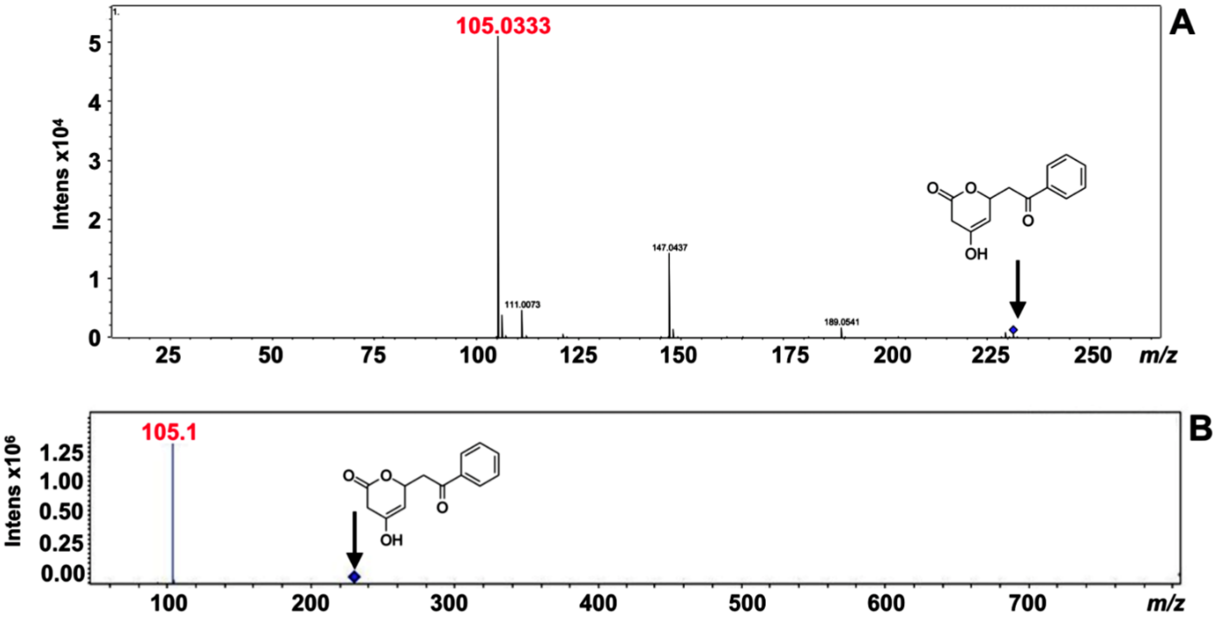

Figure S7. The MS/MS spectra of tetraketide lactone. (**A**) The MS/MS spectra of tetraketide lactone obtained from the enzymatic reaction of GmBPS reported in this study; (**B**) The MS/MS spectra of tetraketide lactone reported by Nualkaew et al. 2012 [12].

m/z	Intensity
105.0335	1809
153.0179	12899
231.0646	3009

Figure S8. The raw mass data of 2,4,6-TriHB measured by positive ion mode LC-MS/MS.

m/z	Intensity
145.0288	17212
151.0033	7548
161.0604	1480
185.0597	788
229.0496	788

Figure S9. The raw mass data of 2,4,6-TriHB measured by negative ion mode LC-MS/MS.
